# Insights into breast health issues in women's rugby

**DOI:** 10.1002/ejsc.12128

**Published:** 2024-07-01

**Authors:** Joanna Wakefield‐Scurr, Edward St John, Kilian Bibby, Nichola Renwick, Neal Smith, Samantha Hobbs, Nicola Brown

**Affiliations:** ^1^ Research Group in Breast Health School of Sport Health and Exercise Science University of Portsmouth Portsmouth UK; ^2^ Portsmouth Hospitals University NHS Trust Portsmouth UK; ^3^ Department of Physical Education and Sport Sciences Health Research Institute University of Limerick Limerick Ireland; ^4^ Centre for Health and Applied Sport and Exercise Research University of Chichester Chichester UK; ^5^ Faculty of Sport, Technology and Health Sciences St Mary's University Twickenham Twickenham UK

**Keywords:** gender, health, medicine, performance, team sport

## Abstract

World Rugby^TM^ supports dedicated women's welfare, injury surveillance and medical/technical interventions, yet breast health has received limited attention. This article aims to provide insights into breast health issues in rugby, including breast impacts and injuries. We discuss how breast anatomy and position may be problematic in rugby. Breast volume relates to body size, which may be increasing in women's rugby, suggesting increased breast surface area and mass, potentially increasing injury risk. Breast health issues in rugby have been reported previously, with 58% of contact footballers (including rugby) experiencing breast injuries. There are damaging effects related to these breast health issues, with breast impacts often causing pain and swelling. Breast impacts may lead to haematomas, cysts and fat necrosis which can calcify over time making them difficult to distinguish from breast carcinoma, causing further investigation and anxiety. In sport, poor bra fit and insufficient support are associated with pain, skin strain and performance decrements. This article reports the potential implications of these breast health issues on performance in rugby. Recent breast‐related projects supported by rugby communities may address recommendations identified in the literature for robust breast injury classifications, updated injury surveillance systems and prospective data collection on breast injury prevalence, severity and impact in rugby. These data should inform breast injury care pathways and intervention research, including evidence‐based bra design. Understanding the implications of breast impacts on tissue properties, health and wellbeing is vital. Finally, data should inform rugby‐specific breast education, raising awareness of this aspect of athlete health.

## Highlights

- This article presents published data suggesting breast impacts and injuries are likely to be prevalent in all codes of women's rugby. Player's body mass may be increasing, which is associated with increased breast volume, surface area and mass, which could have an effect on breast injury prevalence in rugby.
- Breast impacts can induce pain, swelling and haematomas or fat necrosis causing palpable lumps. These impacts can result in cysts that may calcify mimicking breast carcinoma, causing pain, prompting further investigation. Whilst not linked to breast cancer, breast impacts during breast development may cause deformity.
- Published data from bra fittings indicates most contact footballers (including rugby players) wear ill‐fitting bras which are unlikely to protect against the consequences of excessive breast motion; pain, breast skin strain and performance decrements. As body/breast size increases in rugby players, the challenges of finding appropriate bras also increase.
- Previous research suggests breast impacts and injuries are under‐reported and under‐estimated. This article summarises priorities from the literature related to breast health issues in rugby; robust breast injury classifications, updated injury surveillance systems and prospective breast injury data collection, which should inform breast injury care pathways, awareness and intervention research.

## 1 INTRODUCTION

World Rugby^TM^ reports that women and girl's participation in all rugby codes has risen to a quarter of the global rugby playing population (worldrugby.org, 2021). In 2021, World Rugby^TM^ updated their strategic plan to grow participation in women's rugby with the inclusion of dedicated women's player welfare, improved injury surveillance systems and medical and technical interventions for the women's game (Paul et al., 2022). Furthermore, injury was identified as one of three priority research themes by an expert consensus group, alongside women's health and physical performance (Heyward et al., 2021). Despite the increased focus on women's health within all codes of rugby, the breast health of players has been largely neglected. Therefore, the objective of this article is to provide insights on key breast health issues in women's rugby and to identify priority areas for future research. Main focus of the article is breast impacts and injuries, with a secondary focus on breast movement and frictional injuries, and finally the potential influence of these issues on the health and performance of players in all codes of women's rugby.

This article will investigate why breasts might be an issue for women playing contact sports and in particular those playing rugby. It is important to understand the prevalence of breast health issues in women's rugby, and whether these breast health issues have damaging effects on players, as well as negative effects on their performance. This article will review published information on current work regarding breast health issues in women's rugby in order to inform recommendations on future activities to address breast health issues in rugby. Based on this, nine priority areas associated with breast health issues in rugby are identified and presented.

## 2 WHY MIGHT BREASTS BE AN ISSUE FOR WOMEN PLAYING CONTACT SPORTS?

The human female breast contains no muscles, it is a wobbling adipose fibroglandular tissue mass with the perimeter attached via a continuous, irregular fibrous ring that has both fibrous and periosteal attachments to the chest wall (McGhee & Steele, 2023). These attachments limit the translation of the breast across the chest, while the skin and Coopers ligaments provide weak structural support to the tissue mound (Gefen & Dilmoney, 2007). The anatomy of the breast makes it a highly malleable structure and the prominent position makes breasts vulnerable to impacts from hard or fast‐moving objects during contact sports. Although breast skin, subcutaneous adipose, superficial fascia, and fibroadipose tissue provide some protection to the corpus mamma (the delicate functional elements of the breast), they do not protect against high‐energy shear or compressive forces, when tissue might be impacted between the rigid thorax and an offending object (McGhee & Steele, 2023). Such forces could damage the delicate lobules, ducts, alveoli, and overlying tissues (McGhee & Steele, 2023). The extensive superficial capillary networks within the breast increase the likelihood of contusions and haematomas from impacts (Holschen, 2004).

The absence of robust anatomical breast support also means that the movement of the body, particularly during dynamic contact sports, can cause excessive breast movement (Haake & Scurr, 2010). Breast movement is reported to cause pain (Mason et al., 1999) and potential skin tissue damage (Norris et al., 2020). Frictional breast injuries are also associated with breast movement which is often exaggerated in sport when sweat is present (McGhee & Steele, 2023). Frictional breast injuries occur due to rubbing of skin on skin or from clothing or equipment.

## 3 WHY MIGHT BREASTS BE A PARTICULAR ISSUE FOR WOMEN PLAYING RUGBY?

The physique and body size of rugby players vary, reflecting the demands of each playing position (Harty et al., 2021). In elite rugby, literature consistently demonstrates that forwards are heavier and have greater fat mass when compared to backs, with these differences observed across Rugby Union (Yao et al., 2021), Rugby League (Scantlebury et al., 2022) and Rugby Sevens (Sella et al., 2019). Observations of body mass in elite women Rugby Union players identified a 6.5% increase between 2015 and 2019, despite no change in stature; the greatest changes in body mass were observed among front row players, increasing from 80.9 kg in 2015 to 91.7 kg in 2019 (Woodhouse et al., 2021).

Body mass and fat mass are positively related to breast mass (Brown et al., 2012). These data suggest that heavier women have larger and heavier breasts. Brisbine et al. (2020b) assessed breast volume among contact football players (including rugby players), and despite the popular belief that athletes are small‐breasted, a broad range of volumes were reported (85–1717 mL), with comparable median breast volumes (342 mL) to the general population (367 mL) (Coltman et al., 2017). When considered across 46 different sports, Brisbine et al. (2019) concluded that body mass index (BMI) and breast size were positively associated with breast injuries. With potential increases in body mass of women Rugby Union players observed over time, the risk of breast injuries is likely to increase, with forwards being at greater risk due to greater body mass and fat mass.

Collision frequency in women's rugby has been reported to range from 0.17 to 0.89 collisions per minute (Nolan et al., 2023; Woodhouse et al., 2021), with tackles accounting for up to 62% (Schick et al., 2008) of injuries in women's Rugby Union. The force exerted during collisions has not been explored in women's rugby but has been reported to range from heavy (7 to 8G) to severe (+10G) in men's Rugby Union, with 90% of tackles made between the shoulder and midthoracic region (Coughlan et al., 2011); these upper body tackles are reported as the greatest injury risk (Quarrie & Hopkins, 2008). Tackling within this zone is valued by teams and players for tactical advantages, such as preventing offloads from the ball‐carrier, but is likely to increase the risk of impact to the breasts.

In addition to the loads exerted during collisions, the technique used within tackles and other contact events (rucks) could influence breast impacts, although no research exists in this area. For example, a smother tackle is performed by the tackler using their chest and wrapping both their arms around the ball‐carrier to trap the ball and prevent offloads. The frequency and effect of smother tackles within women's rugby are unclear, although it was reported as the third most used tackle in elite men's Rugby Union (Stokes et al., 2021). These collisions have been linked to impact injuries in the breast region (pectoralis major muscle injury) in men's Rugby Union (Hayashi et al., 2014). In addition, upper body, front‐on shoulder tackles may place the ball‐carrier at greater risk for breast impact, as the breast would be the first point of contact.

Recently, World Rugby (worldrugby.org, 2023b) has endorsed several rugby governing bodies across the world to implement a community level trial reducing tackle height to the base of the sternum. This lowered tackle height is supported by research from men's rugby, primarily at an elite level, and therefore the effect on women's elite and community level rugby is unknown. The base of the breasts or the body of the breasts (if they folded over the base) can be in‐line or below the base of the sternum; consequently, this law change may inadvertently increase breast impact and injury risk. Therefore, any change in tackle height requires research on the effect it might have on women in rugby to prevent any unforeseen increase in injury risk.

Women's Rugby League and Union players are reported to cover in excess of 5 km in a game (Armendáriz et al., 2024). With unsupported breasts moving up to 15 cm with each step and ∼10 cm in an everyday bra, considerable loading on the internal supporting structures of the breast could occur without appropriate external support (which can reduce movement to ∼9 cm) (Scurr et al., 2011). Sports bras are reported to reduce the magnitude of breast movement (Mason et al., 1999), which is driven by the movement of the torso (Haake & Scurr, 2010). Different sports exhibit different torso motion and consequently different breast support requirements (Risius et al., 2015). Risius et al. (2015) reported a need for sports‐specific sports bras, which now exist for some activities, but not for rugby. No studies have been identified that report the breast support requirements associated with rugby‐specific movements or game demands.

As breast volume in women rugby players may be increasing, and greater breast volume has been associated with greater breast mass (Turner & Dujon, 2005), this increase in mass is likely to increase breast movement, movement‐related issues and the requirement for external breast support. However, in individual bra assessments with 112 contact football players (including rugby players), over half wore ill‐fitting bras (Brisbine et al., 2020b). Frictional‐related breast injuries have also been associated with higher BMI and larger breast sizes (Brisbine et al., 2019), suggesting this could be a particular issue in women's rugby. Further research is warranted to understand bra fit issues in women's rugby in order to inform rugby‐specific educational material.

## 4 WHAT IS THE PREVALENCE OF BREAST HEALTH ISSUES IN WOMEN'S RUGBY?

Data on breast impacts and injuries in women's rugby is limited to one study. Brisbine et al. (2020c) surveyed 297 players from the women's Australian Football League (AFL), Rugby League, Union and 7s and reported that 58% experienced breast injuries, and 48% perceived they affected their performance. Further, prospective data is required in this area to understand the severity and impact of breast injuries in women's rugby.

Because of the lack of prospective data on breast injuries in women's rugby, it is tempting to extrapolate injury data from elsewhere on the upper body. Data collected within the Women's Rugby Injury Surveillance Project (WRISP, 2020) found injury incidence to the sternum region of 2.4 injuries per 1000 playing hours, and within England women's Rugby Union, sternum region injuries were rare. Compared to breast injury frequency, which have a high life‐long prevalence in women's rugby, incidence of other upper body injury appears rare, suggesting caution when making inferences about breast injury from other regional injury data.

Despite the breast requiring external support to reduce movement during dynamic activities, the use of appropriate breast support in contact football (including rugby) was reported as low (<50%; Brisbine et al., 2020c). This is not unusual in sporting populations; during individual bra assessments with Great Britain (GB) Olympic athletes, Wakefield‐Scurr et al. (2023) reported that most athletes wore ill‐fitting bras, with 59% requiring bra size adjustment. However, no research exists to inform our understanding of what constitutes appropriate breast support in rugby.

Friction‐related breast injuries are common across active cohorts (Brisbine et al., 2019; Wakefield‐Scurr et al., 2023), although there are no data exclusively in women's rugby. Seventy eight percent of GB Olympic athletes reported sports bras rubbing/chafing (Wakefield‐Scurr et al., 2023). Brisbine et al. (2019) reported frictional breast injuries (caused by clothing) in 20% of athletes across 46 sports.

## 5 ARE THERE DAMAGING EFFECTS RELATED TO BREAST HEALTH ISSUES IN RUGBY?

Breast impacts can lead to pain, tenderness, bruising, haematoma, swelling, scar tissue and lumps. Traumatic breast impacts can lead to haematomas, which should resolve without treatment within 6 weeks. However, haematomas can sometimes develop into longer‐term palpable nodules. These nodules are diagnosed as oil cysts and/or fat necrosis which can become calcified as time progresses (Smith et al., 2018). These lumps are often painful and difficult to distinguish from breast carcinoma (Gökgöz et al., 1998), leading to further investigation, causing anxiety for months following the injury. Often, the diagnosis is informed by the patient's history of breast trauma; however, where diagnostic uncertainty exists, a biopsy may be taken to provide histopathological confirmation (Genova & Garza, 2024). The majority of blunt injuries to the breast, causing bruising, bleeding or fat necrosis will be expected to resolve with conservative management, with intervention or surgery rarely required (Genova & Garza, 2024).

No studies have been identified comparing these benign breast conditions and breast tissue characteristics between contact sports women and the general population or non‐contact sporting populations. The absence of data makes it difficult to determine potential changes in breast tissue characteristics beyond that experienced by the general population. For example, one study in female boxing (Bianco et al., 2011) reported that 14.7% of the cohort showed fibrocystic breast tissue; however, Chen et al. (2018) reported that fibrocystic breast change is extremely common and occurs in 90% of women during their lives. No studies have been identified that have reported breast tissue properties in women playing any type of rugby.

Although some have postulated that breast trauma may be linked to the development of breast cancer (Rigby et al., 2002), the medical literature does not support this, with Cancer Research UK clarifying that injury, trauma or a blow to the breast does not cause cancer. Breast cancer is the second most common cancer, occurring in one out of seven UK women (Cancer Research UK, 2023). Women are advised to be ‘breast aware’, and this means regularly checking the breasts and reporting any changes to a doctor. Women who report breast lumps or changes in breast size, outline or shape are referred to specialist breast units for investigation. There are some data that exist reporting that breast impacts during early breast development can cause subsequent, long‐term breast asymmetry. Scarring can develop within the fibroadipose tissue mound that could impede breast bud development, potentially creating long‐term breast deformity and asymmetry (Jansen et al., 2002).

With the physiological purpose of the breast being to produce milk, in rugby, it is important to consider the potential effects of repeated breast impacts on lactation. However, research in this area is very limited, although blunt breast injuries have been reported that demonstrate milk‐duct injury (Sircar et al., 2010). Breastfeeding athletes should be reassured that participating in sport is not known to affect the quality or amount of breast milk produced and should be encouraged to continue breastfeeding (Bane, 2015). Initiatives to improve education regarding breastfeeding as an athlete should be encouraged. However, more nuanced information regarding breastfeeding and contact sports is probably required. Further discussions around postpartum considerations for return to rugby are presented in this special issue by Donnelly et al.

Excessive breast movement in sport is likely to occur without the use of appropriate external support (which is common in rugby; Brisbine et al., 2020c) and excessive breast movement is associated with breast pain (Mason et al., 1999). Wakefield‐Scurr et al. (2023) reported that 51% of GB Olympic athletes experienced breast pain and Brisbine et al. (2020b) reported that 44% of elite Australian athletes experienced breast pain. The severity of breast pain is often reported on a numerical rating scale, where clinical literature categorises scores above three out of 10 as clinically significant (Ader & Browne, 1997). In 2010, Scurr et al. reported breast pain scores of four out of 10 during running in an everyday bra, which would be deemed clinically significant. More recent studies have reported mode breast pain severity as three out of 10 for GB Olympic athletes (Wakefield‐Scurr et al., 2023). Research on the prevalence and severity of breast pain in women rugby players would inform our understanding of the magnitude of this issue in comparison to other sporting populations.

Excessive breast movement has been associated with strain on breast skin (Norris et al., 2020). Measurements of strain can be used to evaluate the magnitude and reversibility of a tissue's response to external loading. Norris et al. (2020) investigated breast skin strain in dynamic activities similar to those occurring during all rugby codes, reporting that in general, participants (*n* = 39) did not experience damaging skin strains (>60%; Silver et al., 2001) during bare‐breasted standing, walking and running. However, individual data showed three women experienced potentially damaging breast skin strains (>60%) during standing and walking and seven during running (up to 93%). Women with greater BMI, breast volumes and bust circumferences were more susceptible to damaging their breast skin due to increased peak breast skin strains and strain rates (Norris et al., 2020), which may have implications for rugby players if body mass, breast volume and bust circumference increase (Woodhouse et al., 2021). These insights suggest breast and bra education may be beneficial for players and practitioners to understand potentially damaging breast health issues in rugby.

## 6 WHAT ARE THE IMPLICATIONS OF THESE BREAST HEALTH ISSUES ON PERFORMANCE FOR RUGBY PLAYERS?

The limited literature available indicates that breast injuries can have negative consequences for performance in women's contact sports. Brisbine et al. (2020a) reported players modifying running/playing or limiting specific activities (tackles) to avoid further breast injury. Several players reported placing their hands in front of their breasts to avoid breast injuries. Players also reported that their breast injury negatively affected their sporting performance because they were distracted by pain, and the injury made them hesitant to dive or tackle or it was uncomfortable to run (Brisbine et al., 2020a).

Excessive breast movement has been associated with whole body mechanical changes in sport, although there is no research focusing specifically on rugby. Breast mass displacing across the chest wall during activities such as running has the potential to change technique and efficiency (Milligan et al., 2015). Increases in breast movement have been associated with increases in pectoral muscle activity (Milligan et al., 2014), increases in torso, pelvis and arm movement (Milligan et al., 2015), changes in ground reaction forces (White et al., 2009), reductions in stride length (White et al., 2013), increases in breathing frequency (White et al., 2011) and alterations in oxygen consumption (Fong & Powell, 2022), which have all been associated with decrements in performance. Further research is warranted to understand how reducing breast movement during rugby‐specific activities might influence performance.

## 7 WHAT IS HAPPENING NOW REGARDING BREAST HEALTH ISSUES IN WOMEN'S RUGBY?

This article identifies a lack of data on breast health issues in women's rugby. The only data presented comes from a retrospective study by Brisbine et al. (2020a), which reports that the majority of women experienced breast injury/injuries during their playing career. One challenge for the collection of breast injury data is the absence of widely accepted medical classifications for breast injuries in sport (Brisbine et al., 2019). Brisbine et al. (2019) reported that most sports injury research uses narrow definitions of injury, recording only injuries that result in time‐loss from play or that necessitate medical attention, which may not occur with breast injuries. More recently, based on previous breast injury classification systems in other sectors, McGhee and Steele (2023) presented a breast injury classification system and a summary of recommended assessments and treatments, providing a basis for further research in this area.

In 2019, Brown et al. noted that the current injury surveillance form for community‐based Rugby Union was, apart from gender selection, generic and did not include any elements related to women. In sport in general, Moore et al. (2023) reported that breast health issues likely go unreported as, like other body regions, the breast does not have a specific diagnosis category in commonly used coding systems and, until 2020, did not appear in these coding systems at all. Moore et al. consider the specific implications of this in a rugby setting within this Special Issue.

It is speculated that the absence of consensus around breast injury classifications, coupled with the lack of breast injury surveillance processes, has resulted in limited breast injury data and an underestimation of breast issues in rugby. This supports data presented by Brisbine et al. (2020a) that contact football staff perceived only 5% of their players had experienced breast injuries, in contrast to the 58% who reported actually sustaining breast injuries. In a survey of women athletes across 46 sports, Brisbine et al. (2019) stated that only ∼10% reported their breast injury. In sport in general, the reporting of sensitive injuries, such as breast injuries, is hampered by the availability of support staff of the same sex (Drummond et al., 2005), unsupportive sport environments, fear of negative consequences (e.g., not getting selected) (Ekegren et al., 2014), lack of recognition of symptoms or believing the injury is not severe enough to report (Register‐Mihalik et al., 2013).

Awareness around the need for robust women's rugby injury surveillance is increasing and specific research projects have recently been supported by World Rugby^TM^ including the University of Wollongong's project focusing on breast injuries and protection in women's rugby, the Welsh Injury Surveillance in Girls' Youth Rugby project, research at the University of Edinburgh on the efficacy of the World Rugby^TM^ Activate injury prevention programme for women, the injury prevention in female youth Rugby Union: a multi‐site international study led by the University of Calgary and a cross‐sectional study investigating retired elite female rugby players' health led by Western University in Canada (worldrugby.org, 2023a).

As breast impacts are likely to be common in women's rugby, breast padding or protection seems logical, although lacks an evidence‐base (Brisbine et al., 2020c; Wakefield‐Scurr et al., 2023). Current commercially available, rugby‐specific, breast protectors are non‐rigid, foam devices, usually in the form of a vest top, commonly worn over a sports bra. In 2005, Comstock et al. investigated protective equipment used by women in Rugby Union and found that none of the 234 players surveyed wore any type of breast or chest padding. More recently, Brisbine et al. (2020c) reported 17% of women contact football players (including rugby players) wore breast protective equipment. Interestingly, only 66% perceived it provided protection against breast injuries. This study concluded that a lack of awareness for breast protection was the biggest barrier to use (Brisbine et al., 2020c). Currently, World Rugby^TM^ is conducting a review of Regulation 12, which refers to player's clothing and equipment. A proportion of this review is on women's equipment to promote player welfare, with a focus on breast health and properly fitting sports bras and padding (worldrugby.org, 2020).

Appropriate sports bras are reported to reduce negative consequences associated with breast movement, such as breast pain (Mason et al., 1999), skin strain (Norris et al., 2020) and performance decrements (Fong & Powell, 2022). But sports bra engagement and adherence require awareness and knowledge. Knowledge of breast health issues, such as breast injuries, breast impacts and breast support requirements in rugby, as in other sports, is likely to be low. Recent attention on these issues has led to some breast education initiatives, for example, England Rugby's RugbySafe programme has recently launched The Women and Girls Health and Welfare toolkit on breast health. This resource provides information on breast health considerations when participating in rugby and includes a sports bra fitting guide.

## 8 WHAT DO WE NEED TO DO NEXT TO ADDRESS BREAST HEALTH ISSUES IN RUGBY?

This article has presented calls from previous literature to establish robust and consistent classifications for breast injuries in sport. These breast injury classifications should then inform appropriate injury surveillance systems, which should be validated and implemented via funded, prospective, injury surveillance projects to provide comprehensive data on the prevalence and severity of breast injuries in women's rugby. These data should facilitate the development of appropriate breast injury care pathways following breast injury. Additionally, the lack of research on the longer‐term implications of breast impacts on tissue properties and athlete health and wellbeing presents an opportunity for future observational studies. The requirement for data on the mechanisms of breast injuries in all rugby codes has also been highlighted, which if borne out, may inform injury mitigation strategies, such as technique/law changes (tackle height) and evidence‐based protective devices for the breast.

This article presents evidence that, if not appropriately supported, breasts are likely to move independently during rugby‐specific movements such as running and jumping. As the movement of the breast is driven by the movement of the torso (unless an external force is applied), appropriate breast support is reported to differ across sports. Understanding the movement of the breast during rugby‐specific activities could help to inform the development of evidence‐based, rugby‐specific breast support products, which do not exist currently.

Background data on breast injury and support requirements should inform credible, evidence‐based education for the whole rugby community, which should encourage players to be aware of and discuss their breast health (including recording breast trauma for future clinical breast examinations). Finally, there is an opportunity for the rugby community to raise awareness of this important aspect of women's health.

## 9 CONCLUSIONS

Retrospective data indicates that breast impacts and injuries are likely to be prevalent in women's rugby. Breast injuries are likely to negatively affect performance; players are unlikely to wear breast protection and there is no evidence that breast padding or protection is effective. Breast impacts may cause pain and bruising, which could progress to fat necrosis, which could lead to complications with future breast screening. Breast injuries are expected to be under‐reported and, therefore, under‐estimated. During dynamic sports like rugby, if breasts are not properly supported, excessive breast movement can cause pain, skin tissue damage and negative performance effects. Players are unlikely to wear appropriate sports bras; furthermore, the challenge of obtaining appropriate and well‐fitted breast support is likely to increase if women rugby players increase in size. Consequently, this article has identified nine priority areas associated with breast issues in rugby, which should be considered across all levels and ages of play:

- Implement robust and consistent classifications for breast injuries in women's rugby.
- Utilise these breast injury classifications to inform appropriate injury surveillance systems, which should be validated and implemented via funded, prospective, injury surveillance projects.
- Develop a comprehensive database on the prevalence, severity, implications and mechanisms of breast injury across all codes of women's rugby.
- Establish appropriate breast injury care pathways following breast injury in rugby.
- Investigate the longer‐term implications of impacts on breast tissue properties and on athlete health and wellbeing.
- Investigate the effect of technique and law changes (e.g., tackle height) on breast injuries in women's rugby.
- Use data on the mechanism of breast injuries in rugby to evaluate the efficacy of current breast padding and protection marketed for women's rugby and to inform the development of evidence‐based breast protection across all levels and sub‐populations in women's rugby.
- Investigate breast movement patterns during rugby‐specific activities to inform breast support design.
- Develop credible, evidence‐based education for the whole rugby community, which increases awareness of breast issues, facilitates discussions between players and supports staff and empowers players to discuss and manage their breast care.

## CONFLICT OF INTEREST STATEMENT

The authors report there are no competing interests to declare.
